# Development of a System Dynamics Model to Guide Retail Food Store Policies in Baltimore City

**DOI:** 10.3390/nu13093055

**Published:** 2021-08-31

**Authors:** Siyao Zhu, Cassandra Mitsinikos, Lisa Poirier, Takeru Igusa, Joel Gittelsohn

**Affiliations:** 1 Department of Civil and Systems Engineering, Johns Hopkins Whiting School of Engineering, Johns Hopkins University, 3400 N. Charles Street, Baltimore, MD 21218, USA; cmitsin1@jhu.edu (C.M.); tigusa1@jhu.edu (T.I.); 2 Global Obesity Prevention Center, Department of International Health, Johns Hopkins Bloomberg School of Public Health, Johns Hopkins University, 615 N. Wolfe Street, Baltimore, MD 21205, USA; lpoirie4@jhmi.edu (L.P.); jgittel1@jhu.edu (J.G.); 3 Center for Human Nutrition, Department of International Health, Johns Hopkins Bloomberg School of Public Health, Johns Hopkins University, 615 N. Wolfe Street, Baltimore, MD 21205, USA

**Keywords:** system dynamics, simulation, staple food, decision making, visualization

## Abstract

Policy interventions to improve food access and address the obesity epidemic among disadvantaged populations are becoming more common throughout the United States. In Baltimore MD, corner stores are a frequently used source of food for low-income populations, but these stores often do not provide a range of affordable healthy foods. This research study aimed to assist city policy makers as they considered implementing a Staple Food Ordinance (SFO) that would require small stores to provide a range and depth of stock of healthy foods. A System Dynamics (SD) model was built to simulate the complex Baltimore food environment and produce optimal values for key decision variables in SFO planning. A web-based application was created for users to access this model to optimize future SFOs, and to test out different options. Four versions of potential SFOs were simulated using this application and the advantages and drawbacks of each SFO are discussed based on the simulation results. These simulations show that a well-designed SFO has the potential to reduce staple food costs, increase corner store profits, reduce food waste, and expand the market for heathy staple foods.

## 1. Introduction

Obesity and other diet-related chronic diseases are at epidemic levels throughout the United States, and some of the highest rates are seen among disadvantaged urban populations [1]. In recent years, many communities have developed and tested different policy strategies, such as sugar-sweetened beverage taxes [2,3,4] and junk food taxes [5] to encourage healthy food demands among consumers, urban farm tax credits [6] to incentivize the construction of new supermarkets in low-income areas [7], and other approaches. While some of these approaches appear to have been successful in modifying the food environment, and have even lead to positive dietary shifts [8,9,10,11,12,13], the impact on obesity has not yet been demonstrated.

Despite the mixed results, policy strategies and interventions are justifiable in the retail food environment [14]. Recent research suggests that retail food environments have a significant effect on diet and the risk of obesity and associated health outcomes of low-income consumers [15,16,17,18,19,20,21,22,23]. Low-income minority groups tend to have greater access to higher-priced, nutrient poor foods [24,25,26,27,28] and reduced access to lower-energy dense foods at retail locations [29,30,31,32]. 

The causes of obesity and related chronic illnesses are multifactorial and complex [33,34,35]. Not only are there multiple causes, operating at different levels, but each of these factors can sometimes influence other factors via feedback loops. Systems science simulation methods offer an approach to understand and meaningfully explore this complexity, and to offer a means of virtually testing and comparing different strategies [36,37].

The Baltimore City food system exemplifies this complexity. In low-income areas of Baltimore, few supermarkets or grocery stores exist and communities are largely served by more than 600 corner stores and nearly the same number of carryout restaurants, dollar stores and pharmacies. Many of these small food sources obtain their products from about a dozen local wholesalers and suppliers, as well as warehouse stores such as Costco and Sam’s Club [38,39]. 

In the fall of 2017, the study investigators were contacted by the Baltimore City Food Policy Director (HF) and her team. The City was considering the development and enforcement of a staple foods ordinance (SFO). A SFO is a law requiring all retail food stores to carry a set variety and depth of stock of foods and beverages, typically healthier products that are not commonly stocked by small urban food stores [40]. Such ordinances are being tested in Minneapolis, Minnesota and Passaic, NJ, but challenges have been experienced related to compliance and enforcement [41,42].

The study team has had more than 15 years of experience working in small retail food stores in urban settings [10,43,44,45,46,47], and are familiar with the perspectives and motivations of small store owners. We felt that a successful SFO in Baltimore would need to clearly address their concerns and be demonstrably profitable, with little waste. A simulation of the SFO in Baltimore should take into account actual costs and concerns of small store owners and their customers.

This paper describes the process by which we developed and refined a system dynamics model to simulate a SFO in Baltimore City. The goals of the model were to help the city government decide on the features of the planned SFO (e.g., specific foods, depth of stock, level of enforcement, etc.), and to help store owners set prices in a manner that would lead to strong sales of these foods, sufficient profitability, and minimal waste.

## 2. Materials and Methods

### 2.1. System Dynamics Models

We use System Dynamics (SD) methods to model the effects of potential SFO policies on corner store food supply, demand, and profit. System Dynamics is a computer-aided method for understanding the behavior of complex systems and may be used to inform policy analysis and decision making [48]. It has been applied to understand complex systems in several fields, including population dynamics, ecology, and economics. SD models can simulate system behaviors including material stocks, flows between stocks, time delays in the system, and internal feedback loops [49]. The process of developing the System Dynamics model to simulate the effects of SFOs is described below.

#### 2.1.1. Step 1: Developing a Visual Model

The goal of this SD model is to simulate the effects of potential SFO policies in order to design an optimal SFO that corner store owners will comply with, and will lead to substantive change to the food environment. We first developed a Visual Model to illustrate relationships between variables that affect the behavior of the system. The Visual Model was developed through a series of meetings between researchers who have developed, implemented and evaluated interventions in Baltimore to improve food access [10,47,50,51], the modeling team, and key staff from Baltimore’s Department of Planning. The Visual Model went through multiple iterations. The model is divided into three dimensions: the retailer dimension, the consumer dimension, and the ordinance dimension, each of which is described below.

##### Retailer Dimension of the SFO Visual Model

**Figure 1:**
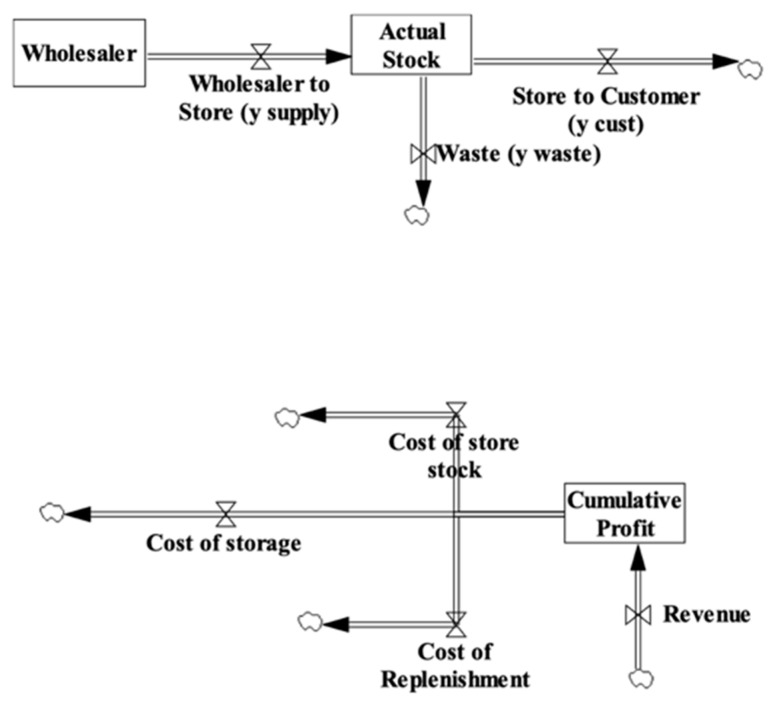
Stocks and flows of food and money within the SFO Visual Model.

The retailer dimension contains elements of the system that the corner store owner controls. This dimension includes two distinct flows: the flow of a particular food (e.g., tomatoes) and the flow of money, which are illustrated in Figure 1.

The flow of food involves the Wholesaler and the Actual Stock, which act as containers that hold food items. The containers are represented as rectangle boxes shown in Figure 1. The corner store can buy food from the Wholesaler’s supply, while the Actual Stock is the supply of food on the corner store’s shelves. Flows are set up to and from these stocks so that food can move through the system. Flows are represented as double-line arrows shown in Figure 1. The Wholesaler to Store flow represents the corner store purchasing food from the wholesaler, the Store to Customers flow represents food that leaves the system when customers purchase it, and the Waste flow represents food that leaves the system when it spoils. The small clouds in Figure 1 represent where the flows leaving the system. Three valves on the flow arrow control the rate at which food moves through each flow in this system.

Money enters the system when customers purchase food, which is represented by the Revenue flow into the Cumulative Profit stock. Money flows out of the system along three cost flows when the corner store pays its operating expenses. The Cost of Store Stock represents the money the corner store pays the wholesaler for food items, while the Cost of Replenishment represents the money the store pays to have food items delivered. The Cost of Storage represents the overhead costs associated with storing food items on the shelves. Four valves control the rate at which money moves through these revenue and cost flows.

**Figure 2:**
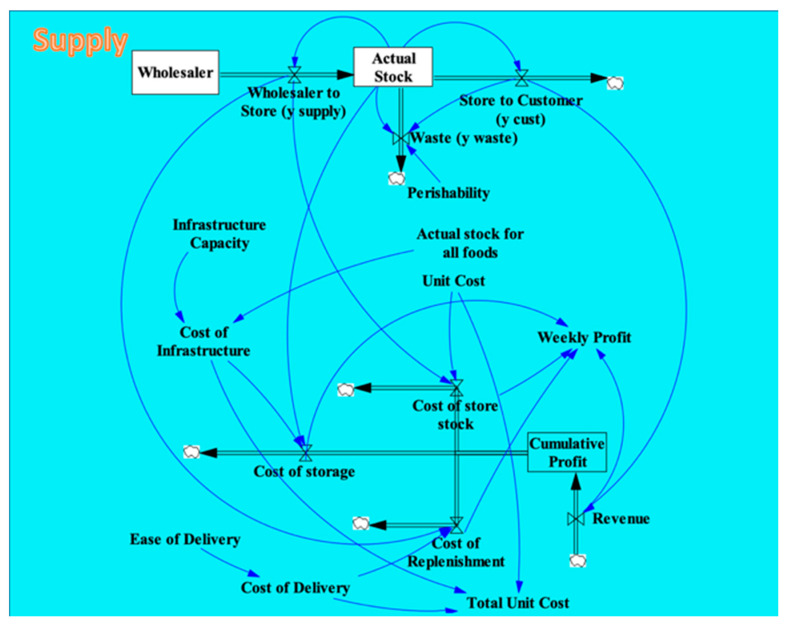
Variables and dependencies within the Retailer Dimension of the SFO Visual Model.

Once the movement of food and money through the system is established, elements that control the flow valves are added. These control mechanisms are represented by arrows that originate from containers, variables, or other valves and point to the valve they affect, such as the blue arrows shown in Figure 2. These arrows represent causal relationships rather than flows of materials.

Within the food flow, the Perishability variable represents the rate at which food spoils and affects the Waste valve. The Waste valve is also controlled by the Actual Stock and the Store to Customers valve, since the food will spoil if the rate at which it is purchased is slow relative to the amount of food on the shelf. The Store to Customers flow valve is controlled by the Actual Stock of food on the shelves, since the customers can only buy as much food as there is in the store.

In the money flow, revenue is generated as customers purchase food, so the Revenue flow valve is controlled by the Store to Customers flow. The corner store must pay the unit price for each food item it buys from the wholesaler, so the Cost of Store Stock valve is controlled by the Unit Cost variable and the Wholesaler to Store valve. Likewise, the store must pay for the delivery of each food item from the wholesaler, so the Cost of Replenishment valve depends on the Wholesaler to Store valve and the Cost of Delivery for each food item. The Ease of Delivery variable affects the Cost of Delivery. The store’s Cost of Infrastructure depends on its Infrastructure Capacity and the Actual Stock for All Foods on the shelves. The Cost of Infrastructure and the Actual Stock factor into the Cost of Storage for each food item. The Total Unit Cost for a food item includes its Unit Cost, the Cost of Infrastructure, and the Cost of Delivery. The Weekly Profit the corner store makes depends on the Revenue flow and the three Cost flows.

##### Consumer Dimension of the SFO Visual Model

**Figure 3:**
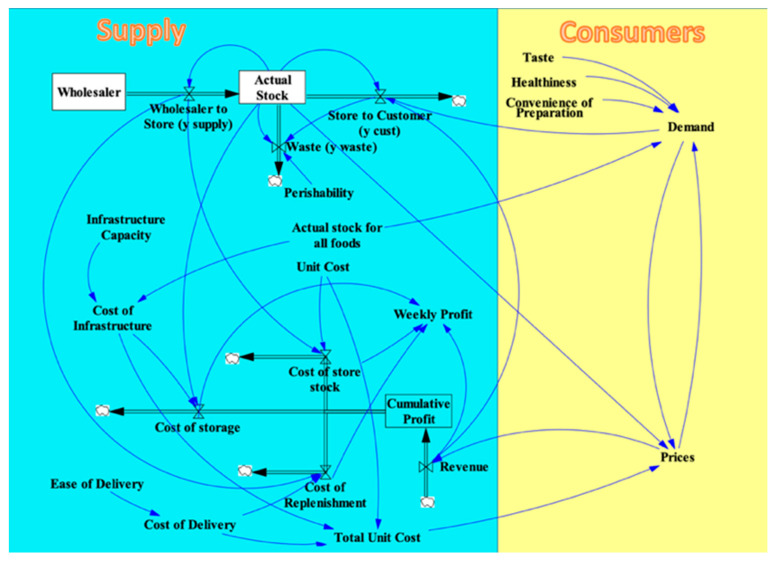
Variables and dependencies within the Consumer Dimension added to the SFO Visual Model.

**Figure 4:**
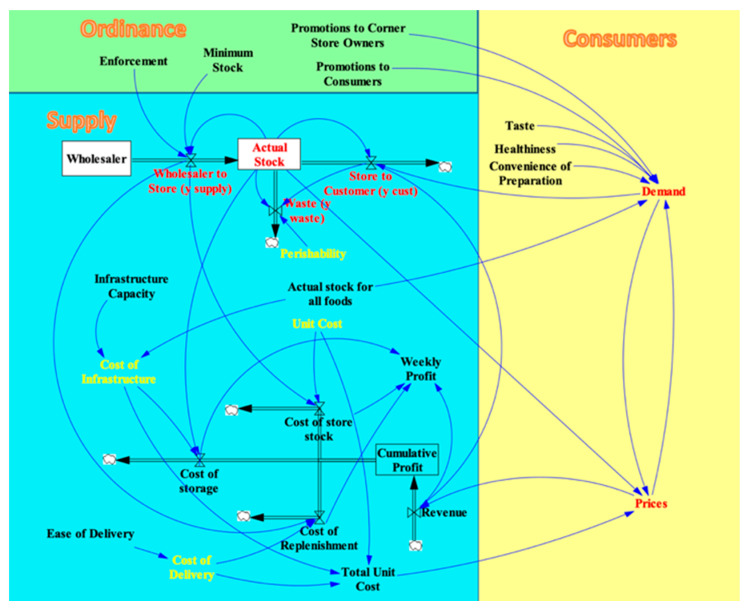
Variables and dependencies within the Ordinance Dimension added to complete the SFO Visual Model.

The consumer dimension contains the elements of the corner store system that consumers influence directly. In this dimension, consumer Demand for a type of food affects that food’s Price, and the Price, in turn, affects Demand, creating a Demand–Price feedback loop. Additionally, the Demand depends on the food’s Taste, Healthiness, and Convenience of Preparation since these features influence a consumer’s decision to purchase food. Demand is also influenced by the Actual Stock for All Foods in the store because a store with a wide variety of foods will attract more customers. The Price of a type of food affects the Revenue flow and depends on the Total Unit Cost and the Actual Stock of that particular food in the store. The variables and controls of consumers dimension of the visual model is shown in the yellow area in Figure 3. To illustrate the relationship between variables such as Price and Demand, a simple Economic Model is built based on the law of demand in microeconomics. The law of demand basically introduced an inverse relationship between price and demand [52]. Several related variables, such as Minimum Stock and Amount Supplied, are added to this Price–Demand relationship to make it fit the needs in our model. The Economic Model is a sub-model of the system consisting of the elements in red and yellow in Figure 4 below and it is explained in greater detail in Appendix B.

##### Ordinance Dimension of the SFO Visual Model

The ordinance dimension contains the elements of the food system that the government seeks to control by introducing an SFO. The Minimum Stock variable represents the amount of a staple food that the SFO requires the corner store to stock, while the Enforcement variable represents how strictly the government enforces this minimum standard. These variables affect the Wholesaler to Store flow in the supply dimension because the corner store must purchase at least the minimum amount of a staple food from the wholesaler. The Promotions to Corner Store Owners variable represents SFO-sponsored training that corner store owners and employees receive to improve store management and comply with the ordinance. The Promotions to Consumers variable represents SFO advertising that influences consumers to buy the staple food products at the store. These two variables affect Demand in the consumer dimension. The variables and controls in ordinance dimension are shown in the green area in Figure 4.

#### 2.1.2. Step 2: Developing the Mathematical Model

The Mathematical Model expresses all of the relationships described by the Visual Model in mathematical language that a computer can use to run simulations. This section describes the development of the SD equation set that runs the model simulations. All of the equations referenced in this section can be found in Appendix A. 

As with the Visual Model, the Mathematical Model is divided into two flows; the flow of food and the flow of money. These flows occur for each of the 22 types of food and the profits are added together to determine the total profit of the system. Tomatoes will be used as the representative food in the following description of the Mathematical Model.

The SD equations consist of six types of variables:

(y) flows of tomatoes (tomatoes/(time step))
(s) stocks of tomatoes (tomatoes)
(c) unit costs associated with tomatoes (dollars/tomato)
(m) flows of money (dollars/(time step))
(x) data inputs rated on a scale from 1 to 5
(p) slope and intersection parameters for each equation found by the data processing techniques described in Section 2.3

The quantity of tomatoes that the corner store needs each week is the maximum of the stock required by the SFO and the customer demand for tomatoes the week before. The corner store purchases y_supply_ from the wholesaler, which is the difference between the quantity of tomatoes needed and the quantity of tomatoes left over from the previous week, with a minimum of zero tomatoes purchased (A1). The store’s weekly stock of tomatoes, y_actual_, is the sum of y_supply_ and the left-over tomatoes (A2). The total amount of food stocked in the store, y_actual_all_, is the sum of y_actual_ for each of the 22 types of food (A3, A4). The quantity of tomatoes that the customers demand this week, y_demand,_ depends on the price of tomatoes, incentives from the SFO, the quality of the tomatoes, and the total amount of food stocked in the store (A5). The quantity of tomatoes that the customers purchase this week, y_cust_, is the minimum of y_actual_ and y_demand_, since customers cannot purchase more tomatoes than those that are stocked (A6). Once all purchases are made, the model calculates how many tomatoes are wasted, y_waste_, which is the difference between the quantity of tomatoes that spoiled this week and the quantity purchased by customers, y_cust_, with a minimum of zero tomatoes wasted (A7). Equation (A7) assumes that older food is always sold before newer food and depends on the particular food’s shelf life. The quantity of left-over tomatoes at the end of the week, y_actual_end_, is the difference between y_actual_ and the tomatoes that left the system when they were purchased, y_cust_, or wasted, y_waste_ (A8).

The flow of money depends on the corner store’s operating costs. The unit cost of delivery, c_delivery_ is a function of the ease of delivery (A9). The unit cost of storage, c_storage_, depends on the store’s infrastructure capacity and the total amount of food stocked in the store (A10). The total unit cost, c_total_, is the sum of c_delivery_, c_storage_, and the unit cost of a tomato from the wholesaler, c_store_ (A11). This total unit cost determines the price of tomatoes for consumers, c_cust_, by using the Economic Model. The money that the store spends on tomatoes each week is found by multiplying these unit costs by the tomato quantities described above: m_supply_ is the product of c_store_ and y_supply_; m_storage_ is the product of y_actual_ and c_storage_; and m_delivery_ is the product of c_delivery_ and y_supply_ (A12, A13, A14). The money the store makes from the sale of tomatoes, m_cust_, is the product of c_cust_ and y_cust_ (A15). The corner store’s weekly profit from tomatoes, m_profit_, is calculated by subtracting the money the store spends on its expenses from m_cust_ (A16).

### 2.2. Data Collection to Parameterize the Model

Research assistants administered surveys to corner store owners and customers to parameterize key variables in the model. A total of 25 store owners were asked Likert scale-style questions for 22 different healthy foods. Questions related to their perceptions of the food’s profitability, shelf life, optimal stock, customer demand, and ease of storage and delivery. All store owner interviews occurred at the corner store. A total of 23 regular store customers were interviewed about their opinions on the food’s price, taste, convenience of preparation, healthiness, and their likelihood of purchasing the food in different settings. Customers were also asked to rate these categories in order of importance to them when they purchase food. Interviews were conducted in a convenience sample occurring at corner stores and Baltimore City recreation centers.

### 2.3. Data Processing

Data from the surveys given to corner store owners and customers were processed to determine the parameters in the SD equations. The processing methods described in this section were repeated separately for each type of food. The following assumptions were made during data processing:

1. The unit delivery cost is 5% of the price [53].
2. The unit storage cost is 10% of the price plus the effect of the Actual Stock for All Foods variable.
3. The maximum price is 200% of the average price and represents when there is no demand for the food because the price is too high.

#### 2.3.1. Linear Regression to Parameterize the SD Equations

Parameters p_10_ and p_11_ are the intersection and slope parameters for the unit cost of delivery equation (A9). Values for these parameters were found by using linear regression, a process in which the parameters of a linear equation are evaluated using a set of ordered pairs of values for the independent and dependent variables. The predictor vector X contains values of the independent variable, which in this case were the survey responses for the Ease of Delivery variable from each corner store. The response vector Y contains values of the dependent variable, which in this case were the survey responses for the Price variable from each corner store. These price values were multiplied by 0.05 to convert them to the unit delivery cost based on Assumption 1 above. The linear regression equation to find parameters p_10_ and p_11_ is:

Y_delivery_ = p_10_ + p_11_ × X_EaseOfDelivery_ (1)

Parameters p_12_ and p_13_ are the intersection and slope parameters for the unit cost of storage Equation (A10). These values were calculated using the same method as above, except in this case the predictor vector X contained the survey results for the Infrastructure Capacity variable for each store and the response vector Y contained survey results for the Price variable for each store multiplied by 0.10 to find the unit storage cost, based on Assumption 2.

#### 2.3.2. Demand Projection to Prepare Survey Data for Multi-Objective Linear Regression

Extra steps were required to process the y_demand_ survey data before calculating the parameters in the demand Equation (A5). Some corner store owners only completed part of the survey and did not provide data on prices or perceived customer demand. Additionally, the preferred amount of stock was nearly constant among store owners, so these data were not useful for regression. This created incomplete data in the store owner dataset that could not be used directly in the Y vector representing y_demand_. The consumer dataset had sufficient demand data, however, so a processing method that combined data from both datasets was used to predict the demand for each food. The demand projection equations referenced below can be found in Appendix D. 

First, a particular store owner’s desired stock was found by taking the minimum of the store owner’s preferred amount of stock and the store owner’s perceived customer demand in the store owner dataset. Store owners generally rated their preferred amount of stock as 5 on a scale from 1 to 5, so since this number was nearly constant, the minimum of the store owner’s preferred stock and their perceived customer demand was used for r_desired_storeOwner_ in Equation (A32). This rating was converted to a quantity of food using Equation (A32) and the Survey Scale to Amount of Stock Conversion Table below (Table 1). The actual consumer demand at a particular store from the consumer dataset, demand_consumer_, was multiplied by the ratio of the average store owner demand to the average customer demand to project demand in Equation (A33). This demand value was entered into the Y vector for each store. For foods that did not have any store owner data to use in Equation (A33), the average of the mean values of store owner demand, μ_demand_storeOwner_, for all other types of food was used to find dmd_proj_.

#### 2.3.3. Multi-Objective Linear Regression to Parameterize the SD Equations

Multi-objective linear regression was used to determine parameters p_1_ through p_6_ because the demand depends on more than one independent variable. This required the creation of an X matrix, where the column vectors contain scaled survey data on employee training, advertising to customers, and the food’s taste, healthiness, and convenience of preparation. Y vector representing demand as dependent variable and the X matrix of independent variables was used in a multi-objective linear regression model to determine p_1_ through p_6_. The multi-objective linear regression equation is:

Y_demand_ = p_1_ + p_2_ × X_training_ + p_3_ × X_signage_ + p_4_ × X_convenience_ + p_5_ × X_taste_ + p_6_ × X_healthiness_ (2)

Once parameters p_1_ through p_6_ were calculated, parameter p_7_ in Equation (A6), which is the slope of the demand–price curve, could be found using the maximum demand and maximum price values, which are associated with the y intersection and the x intersection of the demand–price curve, respectively. The maximum demand was calculated by multiplying parameters p_1_ through p_6_ by five and adding these values together, since five is the maximum scaled value for each independent variable in Equation (A6). The maximum price was calculated by multiplying the price of a food item by two, based on Assumption 3 above. The price was the mean of the prices reported by corner store owners in the surveys or, if there were no price data for a particular type of food, the price estimate came from an online database [54,55]. In this case, the highest reported price was used because food sold at corner stores is typically more expensive than food sold in grocery stores. Parameter p_7_, the slope of the demand–price curve, was then calculated by dividing the maximum demand by the maximum price.

### 2.4. Simulation Method

**Figure 5:**
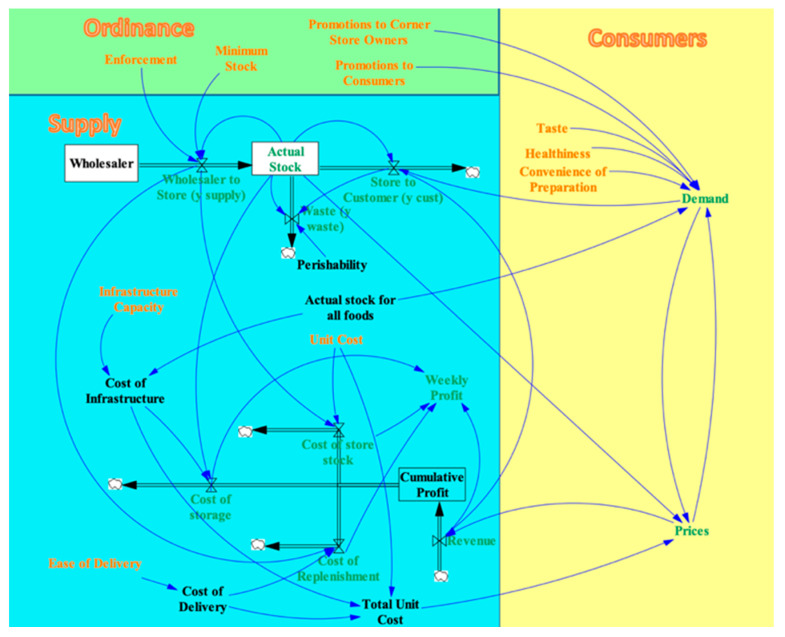
The complete SFO Visual Model with input and result variables highlighted.

There are two types of variables in the simulation:

1. Input Variables, which users enter values for before running the simulation. These variables allow users to adapt the model for their specific environment. These variables are orange in Figure 5.
2. Result Variables, which the simulation reports values for once it has finished. These variables allow users to see how changes to input variables affect the success of the corner store. These variables are green in Figure 5.

#### 2.4.1. Pre-Simulation Profit Maximization

We applied a profit maximization algorithm in order to pre-determine the value of each result variable that produces the optimal profit for the corner store in each time step. Performing the maximization algorithm before the simulation improves computational efficiency. Each result variable is initialized as a two-dimensional matrix with rows that contain the value of the variable as time increases and columns that contain the value of the variable as the price increases. Before the simulation begins, a profit maximization algorithm is run for each time step to determine the optimal price of the food. During the simulation, the variable values associated with the optimal price for each time step will be chosen from the result variable matrices. Further details on the profit maximization algorithm can be found in Appendix C.

#### 2.4.2. Simulation Runs

The simulation is initialized so that the value of each result variable is zero and the minimum stock of each food required by the SFO is also zero. The variable t_ordinance_ tracks when the corner store will be required to stock each type of food. The model requires two time steps for the values of result variables to converge after a new food is introduced, so t_ordinance_ is set so that a new staple food is required by the SFO every two time steps. The simulation also allows the corner store to stock a type of food before the SFO requires it if this generates profit.

The simulation begins in the third time step, because of a two time step delay, and runs for 60 time steps that represent 60 weeks. This time period allows the SFO to set minimum requirements for each of the 22 types of food and allows all of the result variables to converge to equilibrium values by the end of the simulation. In each time step, the simulation runs the 16 equations in the SD equation set separately for each type of food. The equations are always the same, but the parameter values change depending on the survey data for each type of food, as described in Section 2.3. The simulation records the values of the result variables for every food in each time step. The equilibrium value of each result variable at the end of the simulation is the global optimum value for that variable in the system.

#### 2.4.3. User Interface (UI)

**Figure 6:**
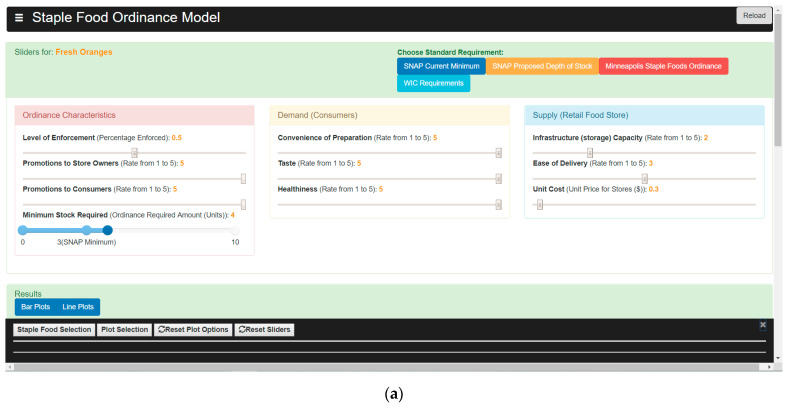

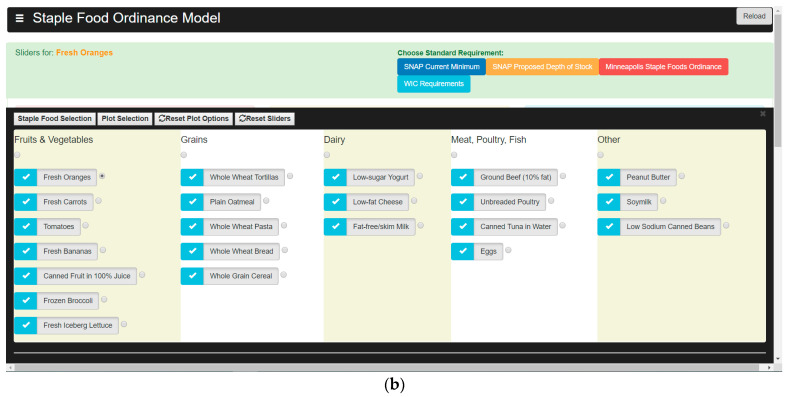
User Interface input options: (**a**) Sliders for input variables, four buttons for the existing sample ordinances; (**b**) Options to show results for specific food groups.

The simulation was coded in JavaScript for easy integration into an HTML web-based application referred to as the User Interface (UI), which users can access to run the model. The UI contains sliders that allow users to set the values of input variables, as seen in Figure 6a,b. Users can set the values for each type of food separately, or they can set the values for an entire food group (e.g., “Grains”). Users can also choose which result variables they would like the UI to display. The results section of the UI can display bar graphs of the optimal variable values in the last time step or time series over the entire simulation. 

Four buttons at the top of the UI allow users to quickly view the simulation results for four sample ordinances that this simulation tested for corner stores in Baltimore. The results of these four simulations are discussed in Section 3. For further details and a link to the UI, see [56].

## 3. Results

Four test simulations of possible SFOs were run to illustrate the results this model can provide. The four simulations developed are referred to as Supplemental Nutrition Assistance Program minimum requirement (SNAP Minimum), SNAP proposed depth of stock (SNAP Depth) [57], Minneapolis SFO [58], and Special Supplemental Nutrition Program for Women, Infants, and Children (WIC) [59], and are presented in greater detail below (Table 2). The sliders for input variables related to customer demand (e.g., Healthiness, Taste) were set to the highest scaled value for all four simulations, while input variables related to SFO requirements (e.g., Enforcement, Minimum Stock) were varied according to the SFO requirements in Table 2. Sample results for each test simulation are provided for four key outputs: weekly profit, weekly stock, customer demand, and recommended sale price.

**Figure 7:**
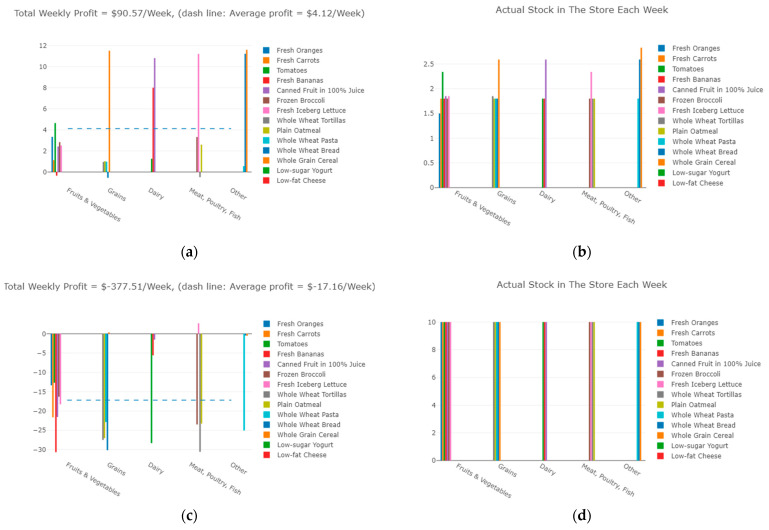

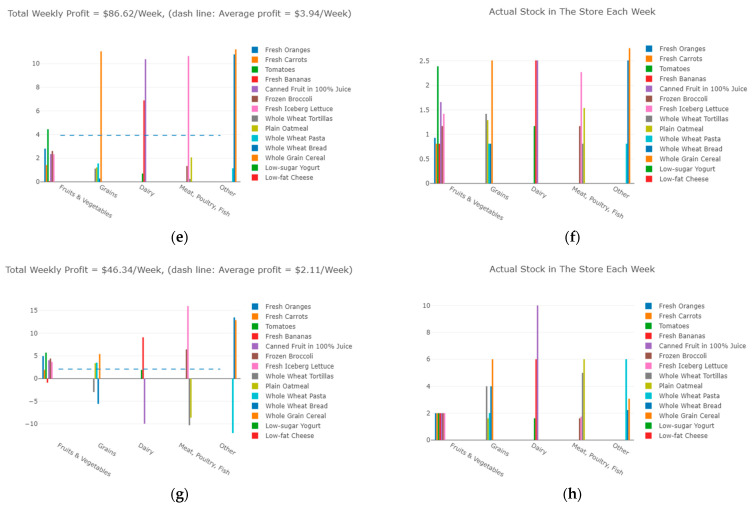
Simulation Results: (**a**) SNAP Minimum Weekly Profit; (**b**) SNAP Minimum Actual Stock each week; (**c**) SNAP Depth Weekly Profit; (**d**) SNAP Depth Actual Stock each week; (**e**) Minneapolis Weekly Profit; (**f**) Minneapolis Actual Stock each week; (**g**) WIC Weekly Profit; (**h**) WIC Actual Stock each week.

The first test simulation was run using SNAP Minimum SFO requirements for the ordinance input variables. The Supplemental Nutrition Assistance Program (SNAP) is a federal anti-hunger program that provides nutrition benefits to low-income individuals and families to supplement their food budget and allow them to purchase healthy and nutritious foods [58]. Stores participating in this program must meet minimum requirements for the amount of staple foods stocked in their store [60]. Figure 7a,b show the results of this SFO simulation. See Table A1 in Appendix E for more detailed numerical results.

The next simulation was run using the SNAP Depth SFO requirements. These requirements come from the same federal program as above, but reflect the fact that in 2016, there was a proposal to raise the minimum stock requirements for staple foods and increase the level of enforcement [61]. These changes were never made. Figure 7c,d show the results of this simulation. See Table A2 in Appendix E for more detailed numerical results.

The third simulation was run using the Minneapolis SFO requirements. The Minneapolis Staple Food Ordinance was the first of its kind in the United States when it was adopted in 2008 and has undergone several revisions since its inception. The ordinance requires grocery stores, corner stores, gas stations, dollar stores, and pharmacies to stock a certain quantity of food items in 10 different categories (e.g., fruits and vegetables, whole grains, etc.) [62]. Figure 7e,f show the results of this simulation. See Table A3 in Appendix E for more detailed numerical results.

The final simulation was performed using WIC SFO requirements. The Special Supplemental Nutrition Program for Women, Infants, and Children (WIC) provides federal grants for supplemental foods, health care referrals, and nutrition education to low-income pregnant women, postpartum women, infants, and children [59]. The simulation results for this program are shown in Figure 7g,h. See Table A4 in Appendix E for more detailed numerical results.

The SNAP Minimum SFO is characterized by low required minimum stocks and moderate enforcement. These requirements resulted in a profitable system that produced the highest weekly profit of the four SFO simulations, as shown in Figure 7a. Most foods yielded positive weekly profits, while only three foods produced negative profits. The majority of the foods have approximately equal levels of actual supply in the store and customer demand, which results in no waste for these foods as shown in Table A1. This resulted in higher profits because no money was wasted by paying supply, delivery, and storage costs for food that spoiled before it was sold. The SNAP Minimum SFO was effective because supply closely followed demand, resulting in high weekly profits.

The SNAP Depth SFO had high required minimum stocks and high enforcement, so the amount of food stocked in the store was much higher than customer demand levels, as seen in Figure 7d. This caused high levels of food waste, as shown in Table A2, which contributed to the negative overall weekly profit in Figure 7c. The SNAP Depth SFO illustrates that restrictive SFOs increase the waste of foods while causing corner stores to lose money, which can reduce compliance with the ordinance.

Though none of the foods in the Minneapolis SFO generated a negative weekly profit, the total weekly profit was slightly lower than in the SNAP Minimum SFO, as seen in Figure 7e. The Minneapolis SFO had moderate required minimum stocks and low enforcement, so the supply of staple foods was entirely driven by customer demand, which results in no waste at all, as shown in Table A3. The more relaxed minimum stock and enforcement levels contributed to lower staple food supply levels, so the Minneapolis SFO facilitates less growth in the staple food market compared to the SNAP Minimum SFO. Another issue with this ordinance should also be considered. Due to very low enforcement of the ordinance, corner store owners may choose not to stock any staple foods in order to increase their profits, which is what actually occurred in Minneapolis [42]. This scenario shows that relaxed SFOs may fail altogether because staple foods are not stocked. 

The WIC SFO produces a positive net weekly profit for the corner store despite the fact that many specific foods produce negative weekly profits, as seen in Figure 7g. The WIC SFO has high levels of enforcement for all foods, but the required minimum stock varies from moderate to high depending on the food, thus allowing the ordinance to “tune” the market by requiring higher stocks for certain targeted foods. This generated higher customer demand for these foods, though the demand was not always high enough to produce a profit. Foods with moderate minimum required stocks could fluctuate more depending on demand, producing higher profits that balanced out the losses of the targeted foods. This allows the corner store to expand the staple food market while still producing a profit. Additionally, by considering the perishability of the different types of food when setting ordinance requirements, even the foods with higher supply than demand result in no waste, as shown in Table A4. 

## 4. Discussion

This paper presents the first simulation of a staple foods ordinance, a new type of policy that is being tested to improve the urban food environment from both supply and demand aspects. Using a group design process and data from Baltimore corner stores and consumers, this study developed and tested a system dynamics model for implementing a staple food ordinance under several different scenarios. The model provided recommendations for which features of an SFO are most effective and sustainable for improving Baltimore’s food system, and these findings were shared with city officials to assist in the development of future food policies. A major strength of the model is that it was parameterized using data from actual corner stores and community members, which enhances its credibility among city policy makers. 

The simulation model was developed through collaboration between city policy makers, community researchers, and modelers. The city policy makers specified the requirements and relevant considerations for developing an SFO model to improve the healthy food environment in Baltimore City and decided to use corner stores as the focus research group. The community researchers investigated the focus research group and collected data for the model based on the specified requirements. The modelers developed the system dynamics model based on the survey results provided by the community researchers. Similar group design processes have been implemented in previous studies. In a study on integrating complex systems methods to improve obesity prevention intervention [63], the community team, called the COMPACT Team, investigated the focus community and provided the necessary information for the modelers, called the SUS Systems Map Team, to develop the complex systems model. In another study [64], the community researchers collected survey data for the modelers to build an agent-based model of the obesity intervention program. This SFO study had an additional group of collaborators, the city policy makers, whose expertise provided the community researchers and the modelers with a clearer understanding of the research purpose and goals. This allowed the community researchers to collect better information for the modelers, which produced a more accurate model of the system. 

Our model provides important insights for city policy makers developing SFOs. The simulations indicated that a heavily prescriptive SFO, such as the SNAP Depth SFO, would be untenable for small food retailers because such a set of requirements results in negative profits and extensive food waste. On the other hand, our model showed that a relaxed ordinance such as the Minneapolis SFO has little effect on the growth of the staple food market. An intermediate SFO that targets specific foods can be used to expand the market for those foods while still maintaining a profitable system with no food waste, as demonstrated by the simulation of the WIC SFO. The varied results for these three scenarios illustrate the importance of effective SFO planning to expand the market for staple foods without resulting in financial losses and food waste.

The sample size of survey data collected from 25 corner store owners and 23 consumers is relatively small, which limits the accuracy of the model. Hence, the model’s accuracy can be further improved by collecting additional survey responses from both corner store owners and consumers. Additionally, while the model is generally designed to fit any corner store scenario, small adjustments are required for the model to work well in different environments. For example, the SD equation parameters should be calculated to reflect the conditions of the specific food environment. This could involve collecting data on the prices of foods from particular wholesalers, incorporating seasonal data into the projected prices of fruits and vegetables, or estimating the overhead costs associated with maintaining a corner store in a particular area, among other characteristics of the specific food environment.

The work presented has several limitations. The simulation model developed is largely from the perspective of small corner store owners. Outcomes are expressed in terms of profits and losses, food waste, and amount of store stock, among other metrics that are important to these particular stakeholders. Additional refinements of the model could be expressed in terms of potential impact on consumer diet or health, for example. A second limitation of this model is the way that ordinance enforcement is characterized. The experience of the Minneapolis SFO showed that lack of consistent enforcement reduced the effectiveness of the ordinance, since policymakers had difficulty reaching a consensus on acceptable and practical ways to enforce the SFO. This shortcoming can be addressed by developing a parameterized sub-model to simulate the effects of several variables related to enforcement and determine an optimal enforcement approach.

## 5. Conclusions

This paper introduced a System Dynamics model that can assist with the development of staple food ordinances in the city of Baltimore. The goal of these ordinances is to increase the availability of staple foods in low-income areas of the city where residents typically purchase food at corner stores that may not stock healthy foods. This System Dynamics model considers several key elements of corner store systems in order to model the complex interactions between corner stores, wholesalers, customers, and government policies. A visual model was presented to qualitatively illustrate the relationships that affect the flow of food and the flow of money in corner store systems. The visual model was adapted to a mathematical model that uses equations to describe the relationships between elements in the system quantitatively. Four simulations of the mathematical model were run through the User Interface to predict the effects of different ordinances on the corner store system. These simulations showed that a poorly designed ordinance may cause corner stores to lose money and waste more food. A well-designed ordinance has the potential to increase corner store profits, reduce food waste, and expand the market for staple foods. This user-friendly System Dynamics model can be developed further to eventually aid policymakers in the ordinance planning process by forecasting the effects of policies on the complex dynamics of the Baltimore food environment.

## Figures and Tables

**Table 1:** Survey Scale to Amount of Stock Conversion Table.

Score	Desired Amount of Stock
1	0 units
2	1–3 units
3	4–6 units
4	7–9 units
5	10 units or more

**Table 2:** Staple Food Ordinance Requirements Table.

SFO Name	Description	Required Minimum Stock	Enforcement
SNAP Minimum	Minimal stocking requirements required to accept SNAP benefits	Low	Moderate
SNAP Depth	Stocking requirements for a food store under the 2016 United States Department of Agriculture proposed enhanced depth of stock requirements	High	High
Minneapolis	Stocking requirements used by the Minneapolis SFO	Moderate	Low
WIC	Stocking requirements if the store participated in the WIC program as a vendor	Moderate to High	High

**Figure A1:**
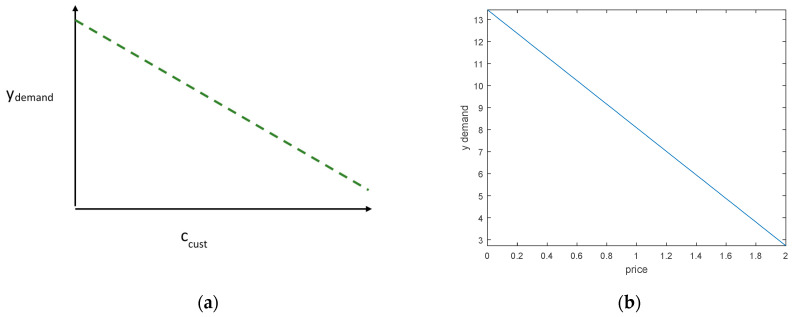
(**a**) Theoretical relationship between price and demand; (**b**) Sample relationship between price and demand using data for “Fresh Oranges”.

**Figure A2:**
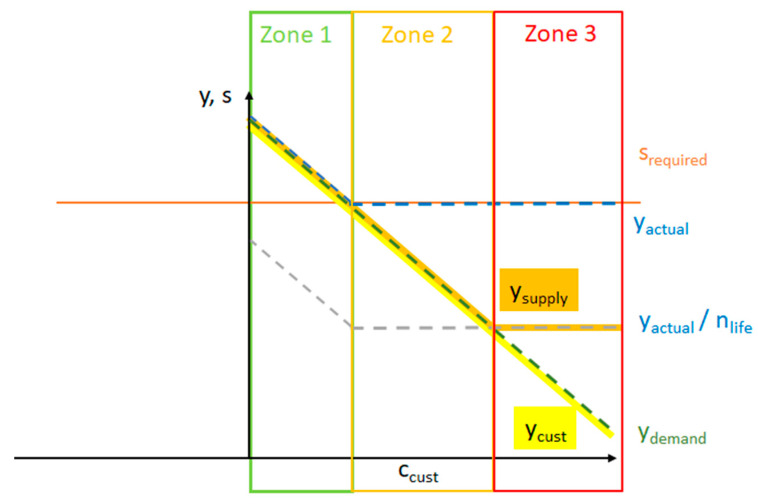
Theoretical relationships between price and amount variables.

**Figure A3:**
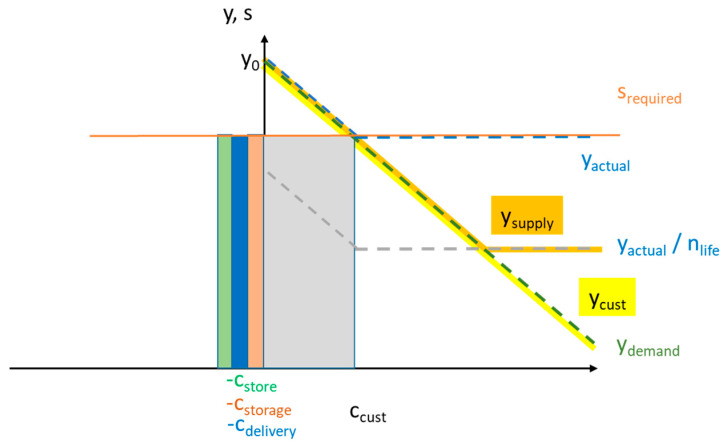
Optimal profit condition in zone 1.

**Figure A4:**
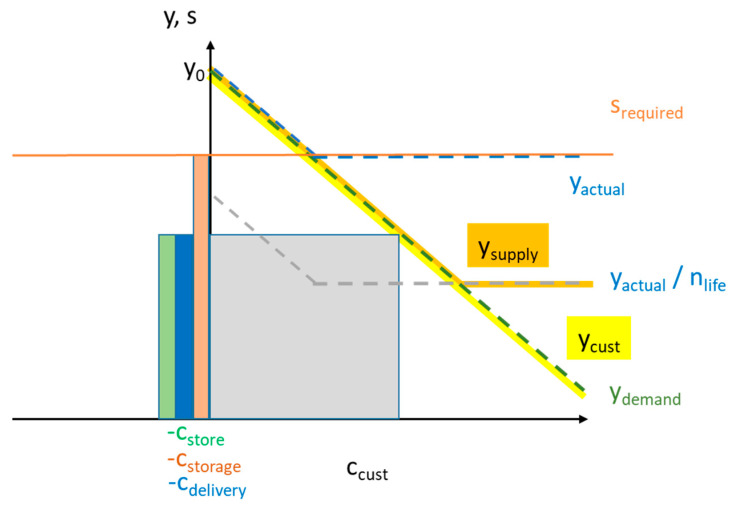
Optimal profit condition in zone 2.

**Figure A5:**
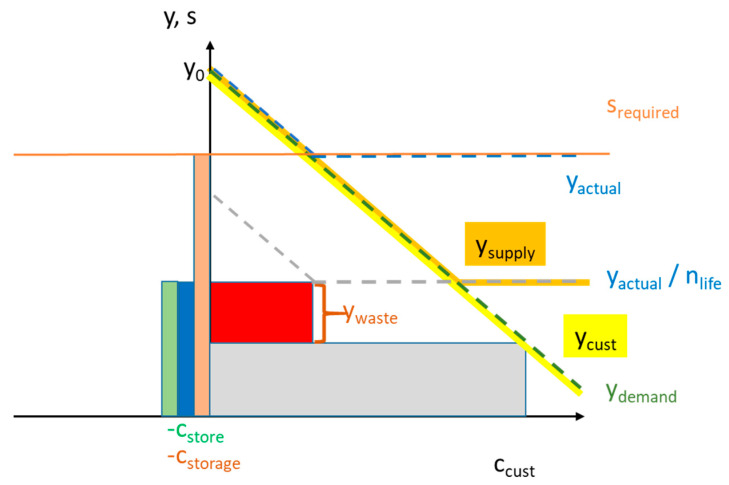
Sub-optimal condition in zone 3.

## Data Availability

Data is contained within the article or supplementary material. The data presented in this study are available in [supplement].

## Supplementary Materials

The following are available online at https://www.mdpi.com/article/10.3390/nu13093055/s1, Table S1: Staple Foods Food Store Owner Survey Data, Table S2: SFO Consumer Data.

## Appendix A. SD Mathematical Equations

The following 16 equations form the SD equation set for the mathematical model. This section describes the variables in each equation in more detail. Variables that are time-dependent use parenthetical notation to denote the time step in units of weeks (e.g., c_storage_(i)). The time step i represents the current time step, “this week,” and the time step i-1 represents the previous time step, “last week”. Variables related to the total amount of all foods stocked in the store are often evaluated in the time step i-2 because the model requires two time steps for these variables to converge after a new food is introduced into the model.

y_supply_(i) = max(s_required_ × x_enforcement_ − y_actual_end_(i-1), y_demand_(i-1) − y_actual_end_(i-1), 0), (A1)

In Equation (A1),

y_supply_ (units/week): Wholesaler to Store, the amount of this food that the wholesaler supplies to the store each week.
s_required_ (units): Minimum Stock of this food as required by the ordinance.
x_enforcement_ (percentage): Enforcement of the ordinance by the government.
y_actual_end_ (units): Actual Stock of this food in the store at the end of the week.
y_demand_ (units/week): Demand for this food by the store’s customers each week.

y_actual_(i) = y_supply_(i) + y_actual_end_ (i-1), (A2)

In Equation (A2),

y_actual_ (units): Actual Stock of this food in the store at the beginning of the week.

y_actual_all-food_(j)(i) = y_actual_(i), (A3)

(A4)
$$y_{\mathrm{actual}\_\mathrm{all}}(i)=\Sigma \;y_{\mathrm{actual}\_\mathrm{all-food}}(j)(i),$$

In Equations (A3) and (A4),

y_actual_all_ (units): Actual Stock for All Foods in the corner store.
y_actual_all-food_ (units): Actual Stock in the store of the specific food (j) in time step (i).

y_demand_(i) = p_1_ + p_2_ × x_training_ + p_3_ × x_signage_ + p_4_ × x_convenience_ + p_5_ × x_taste_ + p_6_ × x_healthiness_ − p_7_ × c_cust_ + p_8_ × y_actual_all_(i-2), (A5)

In Equation (A5),

x_training_ (scale 1–5): Promotion to Store Owner (Training, for example), a rating of the incentives the ordinance provides to promote store compliance.
x_signage_ (scale 1–5): Promotion to Consumer (Signage, for example), a rating of the incentives the ordinance provides to encourage consumers to purchase staple foods at the store.
x_convenience_ (scale 1–5): Convenience of Preparation, a rating of how easy the food is to prepare
x_taste_ (scale 1–5): Taste, a rating of how good the food tastes
x_healthiness_ (scale 1–5): Healthiness, a rating of how healthy the food is
c_cust_ ($/unit): Price for customers to purchase one unit of this food from the store.
p_1_–p_6_: Parameters for the demand equation found by running multi-objective linear regression on the data.
p_7_: Slope of the Demand Curve based on price-demand data.
p_8_: Parameter for the Actual Stock of All Foods that affects the demand for each food.

y_cust_(i) = min(y_demand_(i), y_actual_(i)), (A6)

In Equation (A6):

y_cust_ (units/week): Store to Customers, the amount of food purchased from the store by customers each week.

y_waste_(i) = max((y_actual_(i))/p_9_ − y_cust_(i), 0), (A7)

In Equation (A7),

y_waste_ (units/week): Waste, the amount of food wasted by the store each week.
p_9_: Perishability, the shelf life of each food.

y_actual_end_(i) = y_actual_(i) − y_cust_(i) − y_waste_(i), (A8)

c_delivery_ = p_10_ + x_delivery_ × p_11_, (A9)

In Equation (A9),

c_delivery_ ($/unit): Cost of Delivery, the average cost to deliver a unit of food to the store from the wholesaler.
x_delivery_ (scale 1–5): Ease of Delivery, a rating of how easy it is to deliver the food to the store from the wholesaler.
p_10_: Intersection parameter for the Cost of Delivery equation found by running linear regression on store owner data.
p_11_: Slope parameter for the Cost of Delivery equation found by running linear regression on store owner data.

c_storage_(i) = p_12_ + p_13_ × s_infra_ + p_14_ × y_actual_all_(i-2), (A10)

In Equation (A10),

c_storage_ ($/unit): Cost of Storage, the cost to store a unit of food in the store, includes the cost of shelf space, refrigeration, electricity, and other overhead costs.
s_infra_ (scale 1–5): Infrastructure Capacity, a rating that measures the available space in the store to store food.
p12: Intersection parameter for the Cost of Storage equation found by running linear regression on store owner data.
p13: Slope parameter for the Cost of Storage equation found by running linear regression on store owner data.
p14: Parameter for the Actual Stock of All Foods that affects the unit cost of storage for each food.

c_total_(i) = c_delivery_ + c_storage_(i) + c_store_, (A11)

In Equation (A11),

c_total_ ($/unit): Total Unit Cost for the corner store to purchase the food from the wholesaler, including supply, delivery, and storage costs.
c_store_ ($/unit): Cost of Store Stock, the cost for the corner store to purchase a unit of food from the wholesaler.

m_supply_(i) = c_store_ × y_supply_(i), (A12)

m_storage_(i) = c_storage_(i) × y_actual_(i), (A13)

m_delivery_(i) = c_delivery_ × y_supply_(i), (A14)

m_cust_(i) = c_cust_(i) × y_cust_(i), (A15)

m_profit_(i) = m_cust_(i) − m_supply_(i) − m_storage_(i) − m_delivery_(i), (A16)

In Equations (A12)–(A16),

m_profit_ ($/week): Weekly Profit, the net amount of money the store earns after deducting operating costs.
m_storage_ ($/week): Weekly Cost of Storage to store food in the corner store.
m_supply_ ($/week): Weekly Cost of Store Stock to purchase food from the wholesaler.
m_delivery_ ($/week): Weekly Cost of Replenishment to deliver food to the corner store.
m_cust_ ($/week): Weekly Revenue, the gross amount of money the store earns from customer purchases before deducting operating costs.

## Appendix B. Economic Model

At the core of the mathematical model is the economic model, which is used to understand the relationship between the price of food and the customer demand for food, as expressed in Equation (A5). A demand curve is a concept from basic economic theory that relates price and demand. This model assumes a linear relationship between price and demand, so the demand curve is expressed mathematically as:

y_demand_ = y_0_ − c_cust_/*a*, (A17)

In this equation, c_cust_ is the price of the food, *a* is the slope parameter of the curve, and y_0_ is the maximum demand value at the point where the demand curve intersects the vertical demand axis. The theoretical relationship between price and demand is illustrated in Figure A1a.

We incorporate the demand curve into the model by combining Equation (A5) with Equation (A17), which yields the following:

y_0_ = p_1_ + p_2_ × x_training_ + p_3_ × x_signage_ + p_4_ × x_convenience_ + p_5_ × x_taste_ + p_6_ × x_healthiness_ + p_8_ × y_actual_all_(i-2), (A18)

*a* = 1/p_7_, (A19)

Figure A1b shows a sample relationship between price and demand using Equations (A18) and (A19) with “Fresh Oranges” data.

The economic model maximizes the corner store’s profit in each time step while accounting for the effects of ordinance requirements, customer demand, and food waste. Figure A2 illustrates this graphically by plotting amounts of food as a function of price in a given time-step. The units of all the y variables and s variables are constant amounts of food independent of time.

The green dashed line in Figure A2 shows that as price increases, y_demand_ decreases [65]. In zone 1, y_demand_ is greater than the minimum stock required by the ordinance, s_required_, so the amount of food stocked in the store, y_actual_, is the same as the demand. In zone 2, the demand drops below the minimum required stock so the actual amount of food stocked in the store will be the same as the minimum required stock. The system is in equilibrium when supply equals demand, so both y_supply_, the amount of food supplied by the warehouse, and y_cust_, the amount of food purchased by customers, decrease as demand decreases to maintain equilibrium in zones 1 and 2 [65].

The grey dashed line in Figure A2 plots the amount of food that spoils in each time step as a function of price. The amount of food that spoils is equal to y_actual_/n_life_, where n_life_ is the shelf life of the food. If y_demand_ is greater than the amount of food that will spoil in this time step, then no food is wasted because all of it is purchased by customers. If y_demand_ drops below the amount of food that will spoil, as in zone 3, then the wasted food will need to be replenished by y_supply_. The plot for y_supply_ remains constant after this point because it is equal to the amount of food that spoils in this time step.

The three zones in Figure A2 represent three possible scenarios for the movement of food through the corner store system. The processes for maximizing profit in each scenario are described below.

In zone 1, the demand is higher than the minimum stock required by the ordinance, so the actual stock in the store will equal the demand in order to maximize profit. This means that all of the food purchased from the wholesaler will become the actual stock of food in the store, and all of the store’s stock will be sold to customers in order to meet the demand. Mathematically,

y_actual_ = y_cust_ = y_supply_ = y_demand_,

Substituting y_demand_ for the other amount variables in Equation (A12) through (A15) and combining these new equations with Equation (A16), the profit can be calculated as:

m_profit_ = (c_cust_ − c_store_ − c_delivery_ − c_storage_) × y_demand_, (A20)

By replacing c_cust_ in Equation (A20) with Equation (A17), Equation (A20) can be rewritten as:

m_profit_ = (*a* × (y_0_ − y_demand_) − c_store_ − c_delivery_ − c_storage_) × y_demand_, (A21)

The derivative of Equation (A21) is calculated with respect to y_demand_ and set equal to zero to calculate the demand associated with the maximum profit:

(dm_profit_)/(dy_demand_) = *a* × (y_0_ − 2 × y_demand_) − c_store_ − c_delivery_ − c_storage_, (A22)

y_demand_ = y_0_/2 − (c_store_ + c_delivery_ + c_storage_)/(2 × *a*), (A23)

The maximum profit occurs when the demand is lower than y_0_/2 because the second term in Equation (A23) is always positive. This means that the profit will increase as demand decreases toward y_0_/2. Assuming that the minimum required stock is greater than y_0_/2, this means that the profit will be maximized when the supply and demand equal the minimum stock because the actual stock cannot drop below this value. The price should be set to the point where the demand curve intersects the constant line for s_required_ to maximize profit. Readers should note that the case when the minimum required stock is less than y_0_/2 was not considered because the goal of a minimum stock is to encourage corner stores to stock healthy foods. If the demand is high enough to begin with, a lower minimum stock will have no effect on the corner store. Figure A3 shows the optimal condition for zone 1. The grey area under the minimum required stock line represents the revenue that the corner store makes, while the green, blue, and orange areas represent the costs associated with purchasing, storing, and delivering the food.

In zone 2, the demand is lower than the minimum stock required by the ordinance but higher than the amount of food that will spoil in this time step. The store will stock the minimum amount of food required by the ordinance and this will be higher than the amount of food that customers demand and purchase. The amount of food the corner store must purchase from the wholesaler is equal to the amount of food purchased by customers because none of the food spoils on the shelves. Mathematically,

y_actual_ = s_required_ > y_cust_ = y_supply_ = y_demand_,

Based on the above relationships, y_demand_ is substituted for y_cust_ and y_supply_ in Equations (A12), (A14), and (A15), while s_required_ is substituted in for y_actual_ in Equation (A13). As in zone 1, these four equations are substituted in for the monetary variables in Equation (A16) and combined with Equation (A17) to yield the following profit equation:

m_profit_ = (c_cust_ − c_store_ − c_delivery_) × y_demand_ − c_storage_ × s_required_
= (*a* × (y_0_ − y_demand_) − c_store_ − c_delivery_) × y_demand_ − c_storage_ × s_required_, (A24)

Taking the derivative of Equation (A24) with respect to y_demand_ and setting it equal to zero yields the demand associated with the maximum profit in zone 2:

(dm_profit_)/(dy_demand_) = *a* × (y_0_ − 2 × y_demand_) − c_store_ − c_delivery_, (A25)

y_demand_ = y_0_/2 − (c_store_ + c_delivery_)/(2 × *a*), (A26)

In this case, c_storage_ is constant with respect to demand because the store always stocks the minimum required stock, so this cost does appear in Equation (A26). The price should be set so that demand satisfies Equation (A26) to maximize profit. Figure A4 shows the optimal condition for zone 2.

In zone 3, demand is lower than both the minimum required stock and the amount of food that will spoil in this time step. As in zone 2, the actual stock will be equal to the minimum required stock and the amount of food purchased by customers will equal the demand. The amount of food purchased from the wholesaler will now be greater than the amount of food purchased by customers because the store must replenish the additional food that is wasted. Mathematically,

y_cust_ = y_demand_ < y_supply_ < y_actual_ = s_required_,

Here, y_supply_ will be exactly equal to the amount of food that spoils in this time step, regardless of whether or not some of this food is purchased by customers:

y_supply_ = y_actual_/n_life_,

Following the same substitution process as in the other two zones:

m_profit_ = c_cust_ × y_demand_ − (c_store_ + c_delivery_) × y_supply_ − c_storage_ × s_required_
= (*a* × (y_0_ − y_demand_)) × y_demand_ − (c_store_ + c_delivery_) × y_supply_ − c_storage_ × s_required_, (A27)

Taking the derivative with respect to y_demand_ and setting this equal to zero yields the demand associated with the maximum profit in zone 3:

(dm_profit_)/(dy_demand_) = *a* × (y_0_ − 2 × y_demand_), (A28)

y_demand_ = y_0_/2, (A29)

In this case, c_delivery_ and c_store_ are both constant with respect to y_demand_ because the store must always purchase the amount of food that is wasted, which is a constant. Assuming that y_waste_ > y_0_/2, the profit will increase as demand approaches y_0_/2, so the optimal demand occurs just before leaving zone 3, that is, when the demand is exactly equal to the amount of food that will spoil. Figure A5 shows a potential condition in zone 3, rather than the optimal one, to illustrate the effects of y_waste._

## Appendix C. Profit Maximization Algorithm

1. Starting from zero, increase the price to the maximum price over n equal price-steps. Here, n was set to be 50 price-steps and a was set to be 2. The step size was determined using the equations below.
  price_max_ = y_0_ × *a*, (A30)
  price_step_ = (price_max_)/n, (A31)
  where,
  price_max_ ($): The maximum price, defined as 2 times the average price $y_{0}$. This value is also the demand curve’s intersection with the x axis, representing the price when there is zero demand.
  price_step_ ($): The increase in price during each price-step of the profit maximization algorithm.
2. For each price-step, run the SD equation set for 60 time-steps until the value of the total profit converges.
3. Compare the values of the total profit for each price-step.
4. Save the index of the price-step that result in the highest profit. This index locates the column that will be used for all of the input variable matrices in that time step when the simulation is run.

## Appendix D. Demand Projection Equations

demand_storeowner_ = ScaleToUnitTable(r_desired_storeOwner_), (A32)

dmd_proj_ = (demand_consumer_)/μ_demand_consumer_ × μ_demand_storeOwner_, (A33)

where,

r_desired_storeOwner_ (scale 1–5): The survey rating of either the store owner’s preferred amount of stock or the store owner’s perceived consumer demand, whichever is smaller in the store owners’ dataset.
demand_storeowner_ (units/week): The quantity of a particular food that the store owner demands at a particular store in the store owners’ dataset
μ_demand_storeOwner_ (units/week): The mean value of store owner demand across all corner stores for a particular food.
demand_consumer_ (units/week): The amount of a particular food that consumers demand at a particular store in the consumers’ dataset.
μ_demand_consumer_ (units/week): The mean value of consumer demand across all corner stores for a particular food.
dmd_proj_ (units/week): Projected demand data used for linear regression.

## Appendix E. Detailed Numerical Results from SFO Simulations

**Table A1:** Simulation Results for the SNAP Minimum SFO.

Type of Food	Weekly Profit	Actual Stock of Food (Units of Food per Week)	Waste (Units of Food per Week)
Total	$90.57	43.93	0
Average	$4.12	2.00	0
Fresh Oranges	$3.34	1.50	0
Fresh Carrots	$1.12	1.80	0
Tomatoes	$4.65	2.34	0
Fresh Bananas	−$0.35	1.80	0
Canned Fruit	$2.42	1.85	0
Frozen Broccoli	$2.84	1.80	0
Fresh Iceberg Lettuce	$2.52	1.85	0
Whole Wheat Tortillas	$0.96	1.85	0
Plain Oatmeal	$1.03	1.80	0
Whole Wheat Pasta	$0.98	1.80	0
Whole Wheat Bread	−$0.55	1.80	0
Whole Grain Cereal	$11.52	2.59	0
Low-Sugar Yogurt	$1.26	1.80	0
Low-Fat Cheese	$8.01	1.80	0
Fat-Free/Skim Milk	$10.82	2.59	0
Ground Beef (10% fat)	$3.32	1.80	0
Unbreaded Poultry	$11.22	2.34	0
Canned Tuna	−$0.49	1.80	0
Eggs	$2.60	1.80	0
Peanut Butter	$0.55	1.80	0
Soymilk	$11.23	2.59	0
Canned Beans	$11.61	2.83	0

**Table A2:** Simulation Results for the SNAP Depth SFO.

Type of Food	Weekly Profit	Actual Stock of Food (Units of Food per Week)	Waste (Units of Food per Week)
Total	−$377.51	220	2.61
Average	−$17.16	10	0.12
Fresh Oranges	−$13.32	10	0.56
Fresh Carrots	−$21.62	10	0.58
Tomatoes	−$12.67	10	0.52
Fresh Bananas	−$30.67	10	0.47
Canned Fruit	−$21.53	10	0
Frozen Broccoli	−$16.28	10	0
Fresh Iceberg Lettuce	−$18.27	10	0
Whole Wheat Tortillas	−$27.49	10	0
Plain Oatmeal	−$27.04	10	0
Whole Wheat Pasta	−$22.84	10	0
Whole Wheat Bread	−$30.14	10	0
Whole Grain Cereal	$0.37	10	0
Low-Sugar Yogurt	−$28.32	10	0
Low-Fat Cheese	−$5.60	10	0
Fat-Free/Skim Milk	−$1.53	10	0
Ground Beef (10% fat)	−$23.48	10	0
Unbreaded Poultry	$2.72	10	0.48
Canned Tuna	−$30.57	10	0
Eggs	−$23.26	10	0
Peanut Butter	−$25.02	10	0
Soymilk	−$0.44	10	0
Canned Beans	−$0.52	10	0

**Table A3:** Simulation Results for the Minneapolis SFO.

Type of Food	Weekly Profit	Actual Stock of Food (Units of Food per Week)	Waste (Units of Food per Week)
Total	$86.62	34.09	0
Average	$3.94	1.55	0
Fresh Oranges	$2.81	0.93	0
Fresh Carrots	$1.42	0.81	0
Tomatoes	$4.45	2.39	0
Fresh Bananas	$0.05	0.81	0
Canned Fruit	$2.35	1.66	0
Frozen Broccoli	$2.61	1.17	0
Fresh Iceberg Lettuce	$2.33	1.42	0
Whole Wheat Tortillas	$1.12	1.42	0
Plain Oatmeal	$1.22	1.29	0
Whole Wheat Pasta	$1.55	0.81	0
Whole Wheat Bread	$0.28	0.81	0
Whole Grain Cereal	$11.04	2.51	0
Low-Sugar Yogurt	$0.70	1.17	0
Low-Fat Cheese	$6.89	2.51	0
Fat-Free/Skim Milk	$10.38	2.51	0
Ground Beef (10% fat)	$1.34	1.17	0
Unbreaded Poultry	$10.64	2.27	0
Canned Tuna	$0.25	0.81	0
Eggs	$2.07	1.54	0
Peanut Butter	$1.14	0.81	0
Soymilk	$10.78	2.51	0
Canned Beans	$11.21	2.76	0

**Table A4:** Simulation Results for the WIC SFO.

Type of Food	Weekly Profit	Actual Stock of Food (Units of Food per Week)	Waste (Units of Food per Week)
Total	$46.34	74.91	0
Average	$2.11	3.41	0
Fresh Oranges	$4.95	2	0
Fresh Carrots	$1.94	2	0
Tomatoes	$5.71	2	0
Fresh Bananas	−$0.89	2	0
Canned Fruit	$4.02	2	0
Frozen Broccoli	$4.40	2	0
Fresh Iceberg Lettuce	$3.64	2	0
Whole Wheat Tortillas	−$2.96	4	0
Plain Oatmeal	$3.37	1.62	0
Whole Wheat Pasta	$3.47	2	0
Whole Wheat Bread	−$5.58	4	0
Whole Grain Cereal	$5.40	6	0
Low-Sugar Yogurt	$1.93	1.62	0
Low-Fat Cheese	$9.10	6	0
Fat-Free/Skim Milk	−$9.98	10	0
Ground Beef (10% fat)	$6.42	1.62	0
Unbreaded Poultry	$16.01	1.74	0
Canned Tuna	−$10.31	5	0
Eggs	−$8.64	6	0
Peanut Butter	−$12.03	6	0
Soymilk	$13.49	2.23	0
Canned Beans	$12.89	3.08	0
